# Constitutional activation of IL-6-mediated JAK/STAT pathway through hypermethylation of *SOCS-1* in human gastric cancer cell line

**DOI:** 10.1038/sj.bjc.6602133

**Published:** 2004-08-31

**Authors:** K F To, M W Y Chan, W K Leung, E K W Ng, J Yu, A H C Bai, A W I Lo, S H Chu, J H M Tong, K W Lo, J J Y Sung, F K L Chan

**Affiliations:** 1 Department of Anatomical and Cellular Pathology, The Chinese University of Hong Kong, Prince of Wales Hospital, Shatin, NT, Hong Kong; 2 Department of Medicine and Therapeutics, The Chinese University of Hong Kong, Prince of Wales Hospital, Shatin, NT, Hong Kong; 3 Department of Surgery, The Chinese University of Hong Kong, Prince of Wales Hospital, Shatin, NT, Hong Kong

**Keywords:** IL-6, *SOCS-1*, methylation, STAT3, gastric caner

## Abstract

The interleukin-mediated Janus kinase (JAK)/STAT pathway plays a crucial role in carcinogenesis. Recently, increased STAT3 activity was found in hepatocellular carcinoma and multiple myeloma in which there was silencing of *SOCS-1* (suppressor of cytokine signalling-1) by gene promoter hypermethylation. We investigated the expression level of interleukin-6 (*IL-6*) and *SOCS-1* in gastric cancer cell lines. Expression of *SOCS-1* correlated with *IL-6* level in most of the cell lines, except for AGS cells in which *SOCS-1* was absent despite a high level of *IL-6* production. Methylation analysis by methylation-specific polymerase chain reaction and bisulphite sequencing revealed that CpG island of *SOCS-1* was densely methylated in AGS cells. Demethylation treatment by 5′aza-deoxycytidine restored *SOCS-1* expression and also suppressed constitutive STAT3 phosphorylation in AGS cells. Moreover, methylation of *SOCS-1* was detected in 27.5% (11 of 40) of primary gastric tumours samples, 10% (one of 10) of adjacent noncancer tissues but not in any (zero of nine) normal gastric mucosa. Methylation of *SOCS-1* also correlated with the loss of mRNA expression in some primary gastric cancers. In conclusion, this is the first report to demonstrate that hypermethylation of *SOCS-1* led to gene silencing in gastric cancer cell line and primary tumour samples. Downregulation of *SOCS-1* cooperates with IL-6 in the activation of JAK/STAT pathway in gastric cancer.

Gastric cancer is the second most common cause of cancer death worldwide (Pisani *et al*, 1993). One of the well recognised environmental risk factors for gastric cancer is *Helicobacter pylori* infection (Graham, 1997). Although this bacterium has been classified as a type I carcinogen by the World Health Organisation (Kuipers and Meuwissen, 1996), the mechanisms by which *H. pylori* causes gastric cancer is not fully understood. It is recognised that there is a strong inflammatory response in *H. pylori*-infected gastric cancer tissues. In particular, upregulation of interleukin-6 (IL-6) levels is observed in gastric cancer tissue (Yamaoka *et al*, 1996, 2001). Furthermore, serum IL-6 levels is shown to correlate with prognosis of gastric cancer patients (Wu *et al*, 1996). These data suggest that the activation of IL-6 signalling pathway may be important in the development of gastric cancer.

Interleukin-6 and other interleukin family proteins are thought to be involved in host defense mechanism as well as cancer development (Kabir and Daar, 1995; Wu *et al*, 1996; Schneider *et al*, 2000; Giri *et al*, 2001). The activation of IL-6 signal transduction involves binding to its transmembrane receptor and subsequent activation of the Janus kinase (JAK), which is followed by phosphorylation of STAT (STAT1/3) (O'Shea *et al*, 2002). Phosphorylated STAT protein then translocates into the nucleus with subsequent activation of target genes. One of the STAT-activated genes is *SOCS-1* (suppressor of cytokine signalling-1). Suppressor of cytokine signalling-1 and its family are proteins containing the SH2 domains that interact with JAK and prevent activation of STAT, as well as downregulate the JAK/STAT signalling pathways (Endo *et al*, 1997; Starr *et al*, 1997). Specifically, *SOCS-1* can be rapidly upregulated by IL-6 and is involved in the downregulation of the IL-6-induced activation of STAT3 (Starr *et al*, 1997; Nicholson *et al*, 1999).

Recent findings suggest that the inactivation of *SOCS-1* was one of the targets in cancer development. *Suppressor of cytokine signalling-1* is downregulated by methylation of the CpG island in human hepatocellular carcinoma (HCC), multiple myeloma and pancreatic ductal neoplasm (Yoshikawa *et al*, 2001; Nagai *et al*, 2002; Fukushima *et al*, 2003; Galm *et al*, 2003; Okochi *et al*, 2003). On the other hand, restoration of *SOCS-1* suppressed growth in HCC cell lines and oncogene-activated haematopoietic cells (Yoshikawa *et al*, 2001; Rottapel *et al*, 2002). Taken together, these data suggest that *SOCS-1* functions as a tumour suppressor in the JAK/STAT pathway.

In the present study, we found that there was downregulation of *SOCS-1* gene in gastric cancer cell line AGS due to gene promoter hypermethylation. Furthermore, demethylation treatment by 5′aza-deoxycytidine (5′azaDC) not only restored *SOCS-1* expression in AGS cell but also suppressed constitutive STAT3 phosphorylation. Methylation of *SOCS-1* was detected in 27.5% (11 of 40) of primary gastric tumours samples. We further showed that downregulation of *SOCS-1* correlated with the methylation status in primary gastric cancer. This study provides evidence that the activation of JAK/STAT pathway by aberrant *SOCS-1* methylation in gastric cancer.

## MATERIALS AND METHODS

### Gastric cancer cell lines and tissues

Gastric cancer cell lines AGS (CRL-1739), SNU-16 (CRL-5974), KATO III (HTB-103), and NCI-N87 (CRL-5822) were obtained from the American Type Culture Collection (ATCC, Rockville, MD, USA), while MKN28 (RCB1000) and MKN45 (RCB1001) were obtained from Riken Cell Bank (Tsukuba, Japan). All cell lines except Kato III were grown in RPMI 1640 medium (Gibco BRL, Gaithersburg, MD, USA) supplemented with 10% fetal bovine serum (FBS) (Gibco BRL). Kato III was grown in RPMI 1640 medium supplemented with 20% FBS. All cell lines were kept at 37°C in a humidified incubator with 5% CO_2_ in air.

In all, 40 primary gastric tumour samples and 10 adjacent noncancer tissues were obtained in Prince of Wales Hospital, Shatin, Hong Kong. Nine normal gastric mucosa from individuals without gastric cancer were also obtained as control. All patients gave informed consent for obtaining the specimens. The median age of gastric cancer patients at the time of diagnosis was 70 years old (range from 34 to 83). The male to female ratio was 1.5 : 1. The H&E-stained sections were reviewed by a pathologist to confirm the diagnosis. In all, 19 cases were intestinal type, 17 cases were diffuse type, and four cases were mixed-type gastric adenocarcinoma.

### DNA extraction

DNA from formalin-fixed paraffin-embedded sections were extracted using High Pure PCR Template Preparation Kit (Boehringer Mannheim, Indianapolis, IN, USA). For extraction of cell lines DNA, phenol/chloroform method was used. H&E-stained sections from each tumour sample were examined by an experienced pathologist to confirm their histological diagnosis and assess the tumour content. If tumour content was less than 80%, tumour content was enriched by microdissection using a fine needle under a dissection microscope as described previously (Chan *et al*, 2000).

### Methylation-specific polymerase chain reaction (PCR) (MSP) for *SOCS-1*

Extracted DNA was bisulphite modified by CpGenome DNA Modification kit (Intergen, Purchase, NY, USA). The modified DNA was subjected to MSP using specific primers for *SOCS-1* as described previously (Yoshikawa *et al*, 2001). Primer sequences, annealing temperatures and the expected product size were listed in Table 1

**Table 1:** PCR primer sequences used

**Primer**	**Forward primer (5′ → 3′)**	**Reverse primer (5′ → 3′)**	**Annealing temperature (°C)**	**Product size (bp)**
*SOCS-1*				
MSP	M: TTCGCGTGTATTTTTAGGTCGGTC	M: CGACACAACTCCTACAACGACC	60	93
	U: TTATGAGTATTTGTGTGTATTTTTAGGTTGGTT	U: CACTAACAACACAACTCCTACAACAACCA	60	105
Bisulphite sequencing	TGTAGGATGGTAGTATATAATTAGGTGGT	TAATACTCCAACAACTCTAAAAAACAATC	60	471
RT–PCR	CGCCTGCGGATTCTACTG	AGCAGCTCGAAGAGGCAGT	60	227
IL-6^a^	CACACAGACAGCCACTCACCTC	CTCAGGCTGGACTGCAGGAAC	65	495
*β*-actin	GCATTTGCGGTGGACGATGGAGG	GGTCACCCACACTGTGCCCATCTA	65	653

M=methylated primer; U=unmethylated primer; SOCS-1=suppressor of cytokine signalling-1; MSP=methylation-specific PCR; RT–PCR=reverse transcription–polymerase chain reaction; IL-6=interleukin-6.

a Touch-down programme starting from 65°C.

. A measure of 2 *μ*l of bisulphite-modified DNA were amplified in a total volume of 25 *μ*l containing 1 × PCR buffer II (Applied Biosystems, Foster City, CA, USA), 2 mM MgCl_2_, 0.25 mM dNTP, 1 *μ*M of each primer and 1 U of AmpliTaq Gold DNA polymerase (Applied Biosystems, Foster City, CA, USA) at 95°C for 10 min, 38 cycles of 95°C for 30 s, 60°C for 30 s, and 72°C for 30 s, followed by a final extension of 72°C for 10 min. *In vitro* methylated DNA (IVD) (Intergen, Purchase, NY, USA) was used as a positive control for methylation and water was used as a negative control. A measure of 10 *μ*l of PCR products were loaded onto nondenaturing 10% polyacrylamide gels. The gels were then stained with ethidium bromide and visualised under UV illumination.

### Bisulphite sequencing for *SOCS-1*

Bisulphite-treated DNA was amplified using specific primers for exon 1 region of *SOCS-1* gene as reported by Yoshikawa *et al* (Table 1). The PCR products were cloned into Topo TA cloning kit (Invitrogen, Carlsbad, CA, USA). Five randomly picked clones were sequenced using the dRhodamine Terminator Cycle Sequencing Ready Reaction Kit (Applied Biosystems, Foster City, CA, USA). The sequencing products were separated on an Applied Biosystems 377 automated sequencer (Applied Biosystems, Foster City, CA, USA) and analysed using Applied Biosystems sequencing analysis software.

### RNA isolation and reverse transcription–PCR (RT–PCR) for *SOCS-1* and *IL-6*

Total RNA was extracted from frozen human gastric tissues and cell lines by TRIzol reagent (Invitrogen, Carlsbad, CA, USA). Total RNA (2 *μ*g) was reverse transcribed into cDNA by MMLV reverse transcriptase (Invitrogen, Carlsbad, CA, USA). The expression of *SOCS-1* and *IL-6* was examined by PCR using specific primer as listed in Table 1. For amplification of *IL-6*, a touch-down PCR cycle as described by Lin *et al* (2000a) was used. As an internal control, amplification of *β*-actin was performed. The PCR products were electrophoresed on a 1.5% agarose gel stained with ethidium bromide and visualised under ultraviolet illumination.

### IL-6 protein measurement

*In vitro* IL-6 production from AGS gastric cancer cell line was performed as described previously (Hwang *et al*, 2003). AGS (5 × 10^5^ cells ml^−1^) were plated into 24-well plate and cultured for 2 days in triplicate. Interleukin-6 in the supernatant was measured by Quantikine HS human IL-6 immunoassay (R&D systems, Minneapolis, MN, USA) according to the manufacturer's instructions. The ELISA sensitivity of IL-6 is 0.15 pg ml^−1^.

### Demethylation treatment of gastric cancer cells

To determine if *SOCS-1* expression can be restored by demethylating agent, gastric cancer cell lines were subjected to 5′azaDC treatment. Cells were plated and incubated for 4 days with 5 *μ*M of 5′azaDC (Sigma Chemical Co, St Louis, MO, USA).

### IL-6 and anti-IL-6 antibody treatment of gastric cancer cells

After 24 h of serum starvation, 10ng ml^−1^ of recombinant IL-6 (R&D systems, Minneapolis, MN, USA) was then added into AGS cell for 15 min and proteins were extracted for further analysis. For antibody treatment, 10 *μ*g ml^−1^ of anti-IL-6 antibody (R&D systems) was added into the medium for 24 h. Cells were then harvested for protein extraction and determination of the phosphorylation status of STAT3.

### Western blotting analysis for phospho-STAT3 and total STAT3

Cells are washed with PBS and lysed on ice in buffer containing 1% NP-40, 50 mM Tris at pH 8, 150 mM NaCl, 5 mM EDTA, 10 *μ*g ml^−1^ of aprotinin and pepstatin, 100 *μ*g ml^−1^ of PMSF, and 100 mM NaVO_5_. Protein concentration was determined using the Bio-Rad protein assay. Protein (50 *μ*g) are separated on 10% SDS–polyacrylamide gel electrophoresis and transferred to nitrocellulose membrane at 100 V for 2 h at 4°C using Bio-Rad transfer unit. The transfer buffers used are 25 mM Tris, 192 mM glycine, and 20% methanol. After transfer, the blots are blocked in 10% milk (fatty acid free) with TBS-T (0.1% Tween-20, 20 mM Tris, 137 mM NaCl, and 1 M HCl). The blots are then hybridised with anti-phospho-STAT3 or anti-STAT3 polyclonal antibody (Cell Signaling, Beverly, MA, USA) in 1 : 1000 dilution overnight at 4°C followed by washes in TBS-T, and then incubated with horseradish peroxidase-conjugated anti-rabbit IgG (Santa Cruz Biotechnology, Santa Cruz, CA, USA) at 1 : 2000 dilution for 1 h at room temperature. After several washes, blots are developed using ECL Western blotting detection kit (Amersham Biosciences, Uppsala, Sweden).

## RESULTS

### Expression of *IL-6* in gastric cancer cell lines

**Figure 1:**
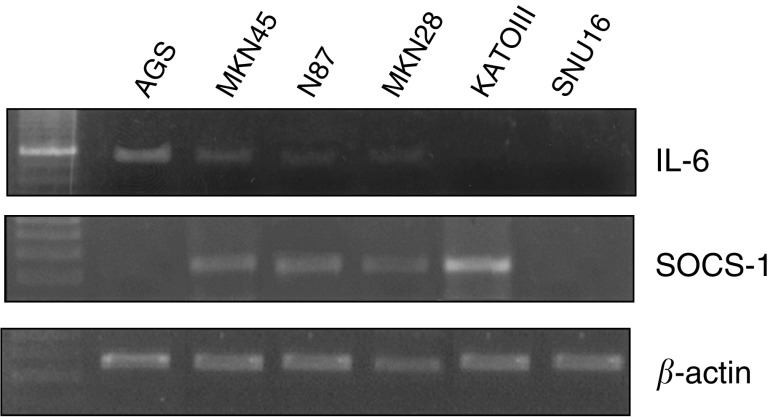
Expression of *SOCS-1* and *IL-6* in gastric cancer cell lines by RT–PCR analysis. *β*-actin was performed as an internal control for loading.

Expression of *IL-6* in six gastric cancer cell lines was assessed by RT–PCR. Our result showed that AGS, N87, and MKN45 expressed IL-6 (Figure 1

, Table 2

**Table 2:** Status of *SOCS-1* and *IL-6* in gastric cancer cell line

	***SOCS-1***		
**Cell line**	**MSP^a^**	**RT–PCR**	***IL-6*^b^**
AGS	M	−	++
SNU-16	U	−	−
MKN28^c^	U	+	+
MKN45	U	+	+
KATO III	U	+	−
N87	U	+	+

SOCS-1=suppressor of cytokine signalling-1; MSP=methylation-specific PCR; RT–PCR=reverse transcription–polymerase chain reaction; IL-6=interleukin-6.

a Result of methylation-specific PCR: M, methylated; U, unmethylated.

b Expression status of IL-6 by RT–PCR.

c IL-6 was weakly expressed in MKN28.

), while MKN28 had barely detectable IL-6 expression. IL-6 expression was absent in KATOIII and SNU16.

### Expression of *SOCS-1* in gastric cancer cell lines

By RT–PCR analysis, *SOCS-1* was expressed in MKN45, N87, MKN28, and KATOIII (Figure 1, Table 2). Since IL-6 and other cytokines are known to upregulate the level of *SOCS-1* gene (Starr *et al*, 1997; Song and Shuai, 1998; Schuringa *et al*, 2000), we also examine IL-6 level in these cell lines. Expression of IL-6 were noticed in MKN45, N87, and MKN28, suggesting that the *SOCS-1* expression may be related to a negative feedback mechanism of IL-6 activation in these cell lines. In SNU16, both *SOCS-1* and *IL-6* was not detected. Although KATO III does not express IL-6, other cytokines may also upregulate *SOCS-1* in this cell line. Notably, despite a high level of IL-6 expression, *SOCS-1* expression was not found in AGS cells (Figure 1).

### IL-6 protein production in AGS cell

To further confirm that IL-6 protein is produced in AGS cells, we examined IL-6 level secreted by AGS cells by high-sensitivity ELISA assay. Our result showed that IL-6 production in AGS cells was 1004±130 ng ml^−1^.

### Methylation of *SOCS-1* in gastric cancer cell lines

**Figure 2:**
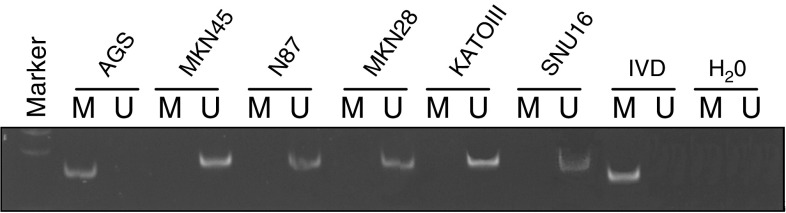
Methylation analysis of *SOCS-1* in gastric cancer cell lines by MSP PCR. U indicates the presence of unmethylated genes; M indicates the presence of methylated genes. *In vitro* methylated DNA (IVD) was used as a positive control for methylation and water (H_2_O) was used as a negative control for PCR.

**Figure 3:**
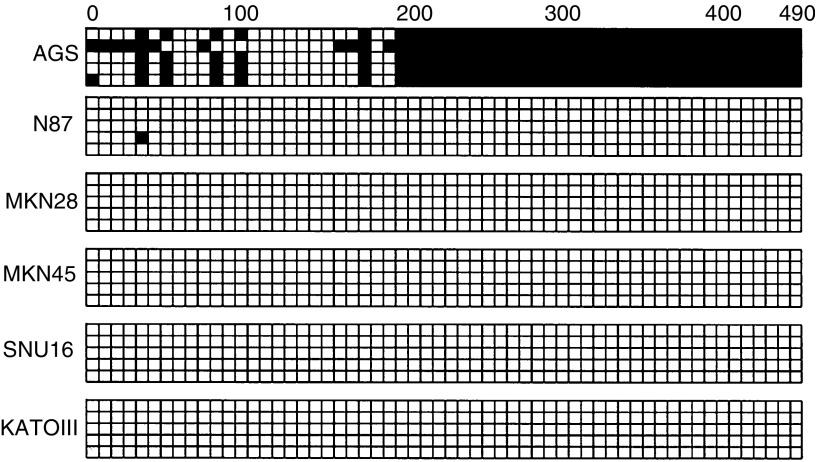
Bisulphite sequencing analysis of six gastric cell lines. Five randomly picked clones of PCR product from bisulphate-treated DNA were sequenced for each cell line. Black and white squares represent methylated and unmethylated CpG site, respectively. The translational start site is indicated as ‘0’.

By MSP among the six cancer cell lines, methylation of *SOCS-1* could only be detected in AGS cell (Figure 2

). Dense methylation pattern in the CpG islands of the exon 1 region of AGS cells was confirmed by bisulphite DNA sequencing, while other cell lines were essentially free of methylation (Figure 3

).

### Demethylation study of *SOCS-1* in the AGS gastric cancer cell line

**Figure 4:**
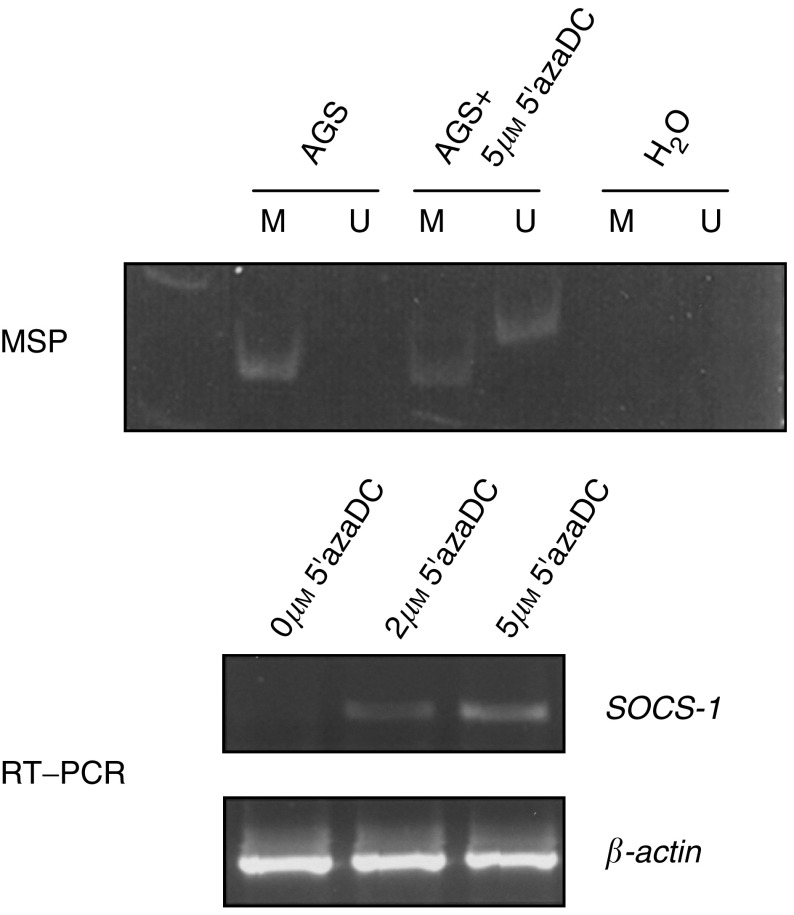
Demethylation treatment of AGS cell line using 5′azaDC. AGS cell was treated with 2 or 5 *μ*M of 5′azaDC for 4 days. The methylation and expression of *SOCS-1* was determined by MSP analysis (upper panel) and RT–PCR (lower panel).

Demethylation study was carried out in the AGS cell. After treatment with 5′azaDC, expression of *SOCS-1* was restored as demonstrated by RT–PCR (Figure 4

, lower panel). Furthermore, MSP detected both methylated and unmethylated allele of *SOCS-1*, indicating that demethylation of the gene occurred (Figure 4, upper panel). These results confirmed that loss of expression of *SOCS-1* in the AGS cell was related to gene methylation.

### STAT3 activation in AGS gastric cancer cell line

**Figure 5:**
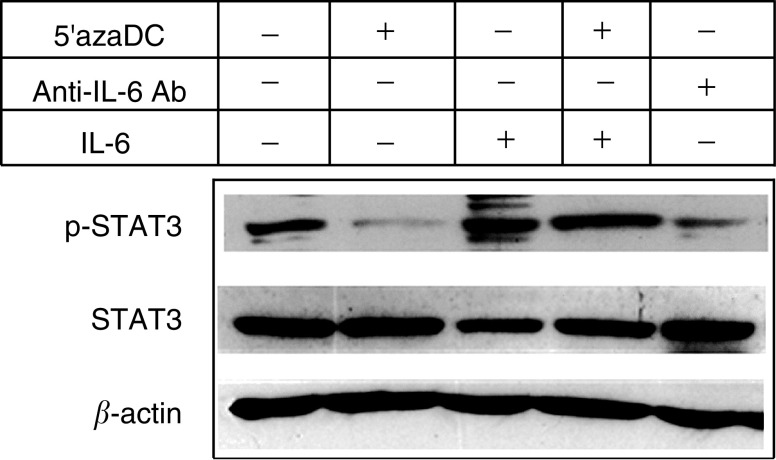
Effect of various treatments on phosphorylation of STAT3 protein in AGS cells. After treatment, cells were harvested for protein extraction and subjected to Western blot analysis with anti-phospho-STAT3 antibody to examine the phosphorylation status of STAT3 protein. As a control, the blot was also probed with total STAT3 and *β*-actin. Starting from left, AGS cells were untreated (panel 1) or treated with 5′azaDC for 4 days (panel 2); recombinant IL-6 for 15 min (panel 3); 5′azaDC for 4 days and then recombinant IL-6 for 15 min (panel 4); or anti-IL-6 antibody for 24 h (panel 5).

Constitutive activation of STAT3 was found in the AGS cells in which IL-6 was highly expressed (Figure 5

, lane 1). We further restored *SOCS-1* expression in AGS cell by treating the cell with 5′azaDC. In addition to the re-expression of *SOCS-1*, it was accompanied by reduction in phosphorylation of STAT3 (Figure 5, lane 2). Pretreatment with anti-IL-6 antibody for 24 h also resulted in inhibition of phosphorylation of STAT3 but not as marked as 5′azaDC treatment (Figure 5, lane 5). Addition of recombinant IL-6 in the medium for 15 min resulted in the reactivation of STAT3 phosphorylation in 5′azaDC-treated AGS cells (Figure 5, lane 4), suggesting that IL-6 was responsible for STAT3 activation. Taken together, these findings suggested that *SOCS-1* play an important role in the inhibition of IL-6-mediated STAT3 activation in AGS gastric cancer cell line.

### Methylation status and expression of *SOCS-1* in primary gastric cancer

**Figure 6:**
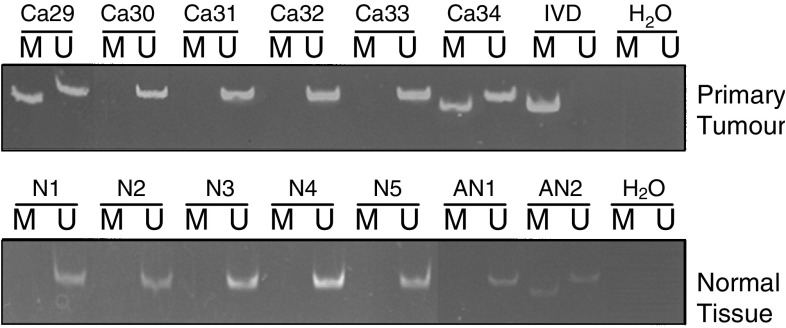
Methylation analysis of *SOCS-1* in primary gastric cancer samples and normal samples by MSP. The upper panel displayed result of cancer samples (Ca) and lower panel displayed the result of normal samples (N) and adjacent noncancer samples (AN).

Methylation of *SOCS-1* was found in 27.5 % (11 of 40) of gastric cancer samples and 10% (one of 10) of adjacent normal mucosa (Figure 6

). Among these 40 gastric cancer samples, no correlation between methylation status of *SOCS-1* with *H. pylori* status, histological type or staging was observed (Table 3

**Table 3:** Association between *SOCS-1* methylation and clinicopathological parameters of gastric cancer

	**Total no.**	***SOCS-1* methylation (%)^a^**	***P*-value^b^**
*Sex*			0.347
Male	24	6 (25.0)	
Female	16	5 (31.3)	
			
*Age (years)*			0.404
⩾60	26	8 (30.7)	
<60	14	3 (21.4)	
			
*H. pylori status*			0.305
Positive	19	4 (21.1)	
Negative	21	7 (33.3)	
			
*Site*			0.510
Antrum	21	6 (28.5)	
Body	11	4 (36.3)	
Cardiac	8	1 (12.5)	
			
*Staging*^c^			0.596
I	7	2 (28.5)	
II	6	3 (50)	
III	19	4 (21.1)	
IV	7	2 (28.5)	
			
*Lauren classification*			0.875
Intestinal type	19	4 (21.1)	
Diffuse Type	17	5 (29.4)	
Mixed type	4	2 (50.0)	

**Figure 7:**
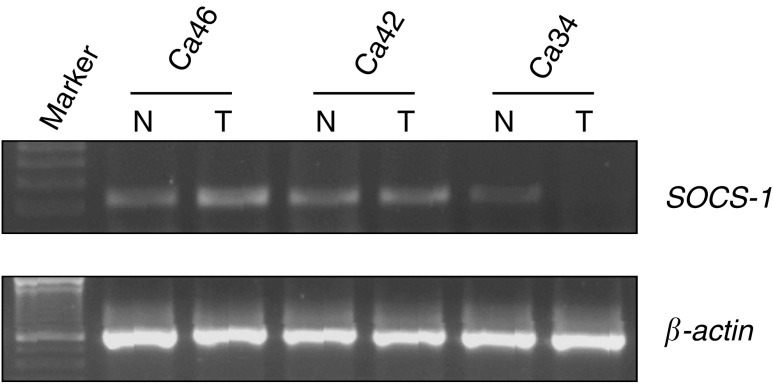
Expression of *SOCS-1* in primary tumour and corresponding normal tissue by RTPCR. Decreased expression of *SOCS-1* was found in tumour samples of case 34 where methylation of *SOCS-1* was also found (Figure 6). T indicates gastric cancer sample and N indicates corresponding normal sample. Expression of *β*-actin was also performed (lower panel) to ensure equal loading.

SOCS-1=suppressor of cytokine signalling-1.

a Number of methylated cases, numbers within parentheses are percentages.

b Comparison were made by *χ*^2^-test or Fisher's exact test (SPSS 10.0).

c Staging according to American Joint Committee on Cancer. Data (staging were not available in one case).

). On the other hand, *SOCS-1* methylation was not found in the normal gastric mucosa from nine individuals without gastric cancer (Figure 6). To further investigate the expression status of *SOCS-1* in these gastric cancer tissues, total RNA from 14 gastric cancer samples and the corresponding normal tissues were extracted for RT–PCR analysis. Downregulation of *SOCS-1* was observed in two (14%) gastric tumour tissues, which also showed *SOCS-1* methylation (Figure 7

, Table 4

**Table 4:** Methylation and expression status of *SOCS-1* in 14 primary gastric cancer samples

			***SOCS-1***	
**Case**	**Type**	**Site**	**Methylation**	**Expression**
Ca21	Intestinal	Antrum	U	NC
Ca22	Intestinal	Cardiac	U	NC
Ca24	Diffuse	Cardiac	U	NC
Ca27	Diffuse	Body	M	Down
Ca28	Intestinal	Body	U	NC
Ca32	Intestinal	Body	U	NC
Ca33	Intestinal	Body	U	NC
Ca34	Mixed	Antrum	M	Down
Ca36	Mixed	Antrum	M	NC
Ca38	Mixed	Cardiac	U	NC
Ca41	Diffuse	Cardiac	U	NC
Ca42	Intestinal	Antrum	U	NC
Ca46	Intestinal	Antrum	U	NC
Ca47	Diffuse	Antrum	U	NC

SOCS-1=suppressor of cytokine signalling-1; M=methylated; U=unmethylated; NC, no change; Down, downregulation.

). These result suggested that methylation of *SOCS-1* gene was responsible for the downregulation of the gene in primary gastric cancer.

## DISCUSSION

Activation of interleukin-mediated JAK/STAT pathway has been recently described to play a crucial role in human cancer development. Constitutive activation of STAT3 has been observed in breast cancer, prostate cancer and leukaemia (Schuringa *et al*, 2000; Lin *et al*, 2000b; Campbell *et al*, 2001; Li and Shaw, 2002). Recent studies also found that blockade of STAT3 activity by the expression of the dominant-negative STAT3 can inhibit growth of AGS gastric cancer cell line, thus further suggesting that JAK/STAT may play an important role in development of gastric cancer (Kanai *et al*, 2003). On the other hand, inactivation of the negative regulator, *SOCS-1*, also leads to the activation of the JAK/STAT pathway. Downregulation of *SOCS-1* by gene promoter hypermethylation has been recently reported in 65% of HCC cell line, 62.9% of multiple myeloma patients samples and 31.6% of pancreatic cancer cell lines with resultant activation of STAT3 (Yoshikawa *et al*, 2001; Fukushima *et al*, 2003; Galm *et al*, 2003). Moreover, restoration of *SOCS-1* suppresses tumour growth in HCC and haematopoietic malignancy (Frantsve *et al*, 2001; Yoshikawa *et al*, 2001; Rottapel *et al*, 2002).

In this study, we have found that *IL-6* was endogenously expressed in several gastric cancer cell lines. Since expression of IL-6 upregulates *SOCS-1*, which participates in the negative regulation of the JAK/STAT pathway (Starr *et al*, 1997; Song and Shuai, 1998; Losman *et al*, 1999; Schuringa *et al*, 2000), we further analysed the expression of *SOCS-1* in these gastric cancer cell lines. Both *IL-6* and *SOCS-1* was not expressed in SUN-16 and the results suggested that this pathway might not be involved in this cell line. Concomitant expression of *IL-6* and *SOCS-1* can be observed in most gastric cancer cell lines, except KATOIII and AGS cell. To further confirm that IL-6 protein is produced in AGS cells, we examined IL-6 level by ELISA assay. IL-6 level of 1004 ng ml^−1^ is found in condition medium of AGS cells, which is consistent with previous report (Crawford *et al*, 2003). Nevertheless, it was interesting to note that *SOCS-1* was not expressed in AGS cells despite the expression of IL-6. We then analysed the methylation status of this gene in AGS cells. Methylation studies by both MSP analysis and bisulphite sequencing confirmed that CpG island of *SOCS-1* was methylated in AGS cells but not other cell lines. Although KATO III did not express IL-6, the high level of *SOCS-1* detected may act as a negative regulation for other cytokines expressed in this cell line (Haque *et al*, 2000; Cottet *et al*, 2001; Naka *et al*, 2001; Eyles *et al*, 2002).

In order to investigate the functional consequences of *SOCS-1* inactivation in JAK/STAT pathway, we analysed the phosphorylation status of STAT3 protein in AGS cells. Under the condition that *SOCS-1* was inactivated by methylation, STAT3 was in hyperphosphorylated state. Restoring *SOCS-1* expression by treating the cells with demethylation agent, phosphorylation of STAT3 was effectively suppressed. On the other hand, blocking the endogenous IL-6 by anti-IL-6 antibody can partially suppress STAT3 activity. Furthermore, addition of recombinant IL-6 restored STAT3 phosphorylation in demethylated-AGS cell, thus suggesting that IL-6 was responsible for STAT3 activation. Taken together, these results suggested that *SOCS-1* was important for the downregulation of JAK/STAT signalling. Methylation-mediated *SOCS-1* inactivation enhanced IL-6-mediated activation of STAT3 in AGS cell.

Moreover, methylation of *SOCS-1* can be detected in about 30% of primary tumour tissues and 10% of adjacent normal tissues. Downregulation of *SOCS-1* was also observed in primary gastric cancer with methylation of *SOCS-1*. Although downregulation of *SOCS-1* was not observed in one sample (Ca36) in which methylation of *SOCS-1* was detected, this discrepancy may be due to heterogeneity of the tumours in which only a small portion of tumour cells have *SOCS-1* methylation. Nevertheless, in this study, we have demonstrated that *SOCS-1* was silenced by hypermethylation in the AGS cell line and a subset of primary gastric tumour tissues. It is also worth noting to point out that hypermethylation of *SOCS-1* was also found in 10% of adjacent normal tissue and this observation suggest that *SOCS-1* methylation may also be involved in the early gastric carcinogenesis process.

Recently, we and others have found that eradication of *H. pylori* in the stomach can reduce the risk of gastric cancer (Uemura *et al*, 1997; Saito *et al*, 2000; Sung *et al*, 2000). The current study may provide clue to the underlying mechanism that *H. pylori*-mediated cytokine expression-enhanced tumour progression in a subset of gastric cancer where *SOCS-1* was hypermethylated. Inhibition of JAK/STAT pathway by demethylation treatment or by applying specific JAK2 inhibitor may open up a new therapeutic strategy against gastric cancer (De Vos *et al*, 2000; Burke *et al*, 2001; Yoshikawa *et al*, 2001).

In summary, loss of expression of *SOCS-1* in AGS cell line was related to gene promoter hypermethylation. This phenomenon together with endogenous *IL-6* expression leads to the activation of STAT3 protein. The increase of STAT3 activity together with overexpression of cytokine in this gastric cancer cell line suggested that alteration of JAK/STAT was important in a subset of gastric cancer.
